# Bayesian nonparametric method for genetic dissection of brain activation region

**DOI:** 10.3389/fnins.2023.1235321

**Published:** 2023-10-18

**Authors:** Zhuxuan Jin, Jian Kang, Tianwei Yu

**Affiliations:** ^1^Department of Biostatistics and Bioinformatics, Emory University, Atlanta, GA, United States; ^2^Department of Biostatistics, University of Michigan, Ann Arbor, MI, United States; ^3^School of Data Science, Chinese University of Hong Kong - Shenzhen, Shenzhen, China; ^4^Guangdong Provincial Key Laboratory of Big Data Computing, Shenzhen, China

**Keywords:** Gaussian process, segmentation, Alzheimer's disease, imaging genetics, PET imaging

## Abstract

Biological evidence indicewates that the brain atrophy can be involved at the onset of neuropathological pathways of Alzheimer's disease. However, there is lack of formal statistical methods to perform genetic dissection of brain activation phenotypes such as shape and intensity. To this end, we propose a Bayesian hierarchical model which consists of two levels of hierarchy. At level 1, we develop a Bayesian nonparametric level set (BNLS) model for studying the brain activation region shape. At level 2, we construct a regression model to select genetic variants that are strongly associated with the brain activation intensity, where a spike-and-slab prior and a Gaussian prior are chosen for feature selection. We develop efficient posterior computation algorithms based on the Markov chain Monte Carlo (MCMC) method. We demonstrate the advantages of the proposed method via extensive simulation studies and analyses of imaging genetics data in the Alzheimer's disease neuroimaging initiative (ADNI) study.

## 1. Introduction

Imaging genetics is an emerging interdisciplinary field with a focus on assessing the impact of genetic variation on brain function and structure. It is a useful tool to uncover the etiologies of complex neuropsychiatric diseases, such as Autism (Ameis and Szatmari, 2012) schizophrenia (Meyer-Lindenberg, 2010) and Alzheimer's disease (Weiner et al., 2013). Traditional genetics studies have attempted to search genetic variants that are strongly associated with a behavior or related phenotypes; however, some findings were weak and inconsistent. There are considerable inter-subject differences in the behavioral measures, usually requiring large sample sizes to detect a signal. For neuropsychiatric disease, many genetic variants may not be directly associated with a clinical outcome or a behavior response but have a strong indirect effect which is mediated through molecular and cellular level information processing by neurons in the brain. We refer to this information processing procedure as brain activity. Functional neuroimaging, including functional magnetic resonance imaging (fMRI) and positron emission tomography (PET), is a set of powerful techniques to indirectly measure the brain activity at each location in the brain. Many current functional neuroimaging studies have focused on detecting the brain activation regions in association with particular cognitive and emotional tasks or at resting state.

Therefore, in imaging genetics studies, it is of great interest is to simultaneously select important genetic variants and detect brain activation regions where the genetic effects are strongly associated with brain activity. We refer to this procedure as genetic dissection of brain activation regions. Our motivating example is joint analysis of the fluorodeoxyglucose positron emission tomography (FDG-PET) data, single nucleotide polymorphisms (SNP) data and clinical data in the Alzheimer's Disease Neuroimaging Initiative (ADNI) study. Alzheimer's disease (AD) is one of the most common neurodegenerative disorders that impair mental functioning. It affects approximately eight percent of people who are 65 years of age or older. It has been shown that AD leads to nerve cell death and tissue loss in the brain (Bookheimer et al., 2000). As AD progresses, the brain shrinks dramatically; and abnormal changes in the brain worsen over time, eventually interfering with many aspects of brain function, such as memory loss, resulting in a decline in some intellectual abilities and changes in personality and behavior. New and potential treatments for AD focus on slowing the progression of the disease, making it important to identify at an early stage markers of future cognitive decline. Genetics studies showed that the presence of some of genes such as APOE and NEDD9 may be associated with cognitive decline in older persons (Wang et al., 2011). Structural magnetic resonance imaging (sMRI) studies (Bookheimer et al., 2000) identified that older persons with normal cognition may show medial temporal atrophy and thus indicate the possibility of future cognitive impairment. Many ADNI studies have focused on the joint analysis of sMRI and SNPs to discover the genetic effects on brain structure (Stein et al., 2010; Zhu et al., 2014; Huang et al., 2015). Functional neuroimaging techniques can facilitate to discover more subtle alternations in brain function as AD progresses, thus analyses of PET or fMRI data in the ADNI studies have drawn much attention recently as well. For example, Huang et al. (2010) and Kundu and Kang (2016) developed statistical methods for leaning the genetic effects on the functional connectivity of AD. In this work, our goal is to study the genetic effects on functional brain activity for people at risk of AD, based on which we can identify the consistent brain activation regions across multiple subjects and quantify the changes of their shapes over times.

Many of them have been adopted to detect the association between imaging biomarkers and genetic variants. The pioneer work includes voxelwise genome-wide association (vGWAS) study (Stein et al., 2010) where each voxel is considered as a phenotype and univariate regression models were fitted for all the combinations of voxels and genetic variants. This approach enjoys the simplicity and fast computations but suffers from the difficulty of the multiple testing problem since the number of voxels often can be up to more than 10,000. To address those limitations, Huang et al. (2015) proposed a joint modeling approach, termed as, Fast voxelwise genome wide association analysis (FVGWAS) with a well family-wise error control procedure and developed efficient computing tools for large-scale imaging genetics studies. Huang et al. (2017) introduced a new framework called Functional Genome-Wide Association Analysis (FGWAS) designed to analyze functional phenotypes like those found in neuroimaging studies. FGWAS improves upon FVGWAS methods by incorporating the unique features of functional phenotypes-like smoothness and correlation-into the statistical model, resulting in more powerful detection of genetic variants affecting brain structure and function. Alternatively, Vounou et al. (2010) and Zhu et al. (2014) proposed to use low rank regression to handle the high-dimensional neuroimaging phenotype, where a latent structure are imposed in the regression coefficients. Besides reduced rank approximation approaches, independent component analysis (ICA) (Liu et al., 2009) and canonical correlation analysis (CCA) (Chi et al., 2013) have been applied to discover the association between the imaging biomarkers and genetic variants with different latent structure assumptions.

Different from all the existing methods, in this work, we propose a Bayesian hierarchical model for genetic dissection of brain activation regions using the level set function with the Gaussian process (GP) prior. We term our method as Bayesian nonparametric level set (BNLS) method. BNLS consists of two levels of hierarchy.

At level 1, a Bayesian nonparametric level set model is developed for characterizing the shape of consistent brain activation regions across multiple subjects. The level set method has been widely used in image segmentation problems (e.g., Balafar et al., 2010; Li et al., 2011; Bergeest and Rohr, 2012), where contours (2D) or surfaces (3D) are represented as the zero-level set of a higher dimensional function, thus spatial voxels can be classified based on the function values: positive (inside the region) or negative (outside the region). We refer to this function as the level set function. The corresponding shape representation can characterize complex topological variations: the appearance of holes or tails, shapes that break down into smaller pieces, etc. The traditional level set based shape estimation problem can be solved by the numerical methods for partial differential equations. In our model, we propose to assign a GP prior to the level set function and make fully posterior inference on the level set function as well as the shape of the activation regions, taking advantages of the good statistical properties of GP.

At level 2, a regression model is adopted to select genetic variants that are strongly associated with the average brain activity within the region over multiple subjects, where a spike-and-slab prior and a Gaussian prior are chosen for feature selection. In particular, we model the average brain activation intensity within the region for each subject as the response variable; and we consider all the genetic variants as well as some clinical factors as predictors. We assign the Bayesian spike and slab prior on the regression coefficients for variable selection and thus to detect the important genetic variants of interest. The spike and slab prior was initially proposed by Mitchell and Beauchamp (1988) and George and McCulloch (1993) and has been broadly adopted for various applications (Chipman et al., 2001; Ishwaran and Rao, 2005a,b). In the spike and slab prior specifications, the coefficients are mutually independent with a two-point mixture distribution made up of a “uniform-like” flat distribution (called “slab”) and a “degenerated-point-mass-at-zero-like” distribution (called “spike”), leading to sparsity in the posterior inference.

Our proposed Bayesian model offers several unique features compared to existing methods. First, the foundational assumptions of our model diverge substantially from those of FVGWAS and FGWAS. Specifically, we focus on probabilistic modeling of brain activation regions and the selection of key genetic variants with significant associations to these regions. Second, we aim for fully Bayesian inferences that account for all sources of variation in the model parameters, which in turn characterize both imaging measurements and genetic variants. Third, our method is designed to efficiently analyze high-resolution images and a moderately large set of genetic variants. It is important to note that we do not expect our Bayesian approach to scale in the same way as variable screening-based methods like FVGWAS and FGWAS. Our model also has the potential to be integrated with prior knowledge-guided Bayesian variable screening methods (He and Kang, 2022). The efficacy of this combined approach certainly warrants future investigation.

The remainder of the manuscript is organized as follows. In Section 2, we present the proposed model with prior specifications, and develop the posterior computation algorithms for fully Bayesian model. In Section 3, we evaluate the performance of the proposed method via extensive simulation studies. In Section 4, we illustrate the proposed method on analysis of the PET and SNP data from the ADNI study to detect influential SNPs and consistent activation regions across subjects. Finally, we conclude our paper by discussion in Section 5.

## 2. Materials and methods

In this section, we develop BNLS: a two-level Bayesian hierarchical model for fitting the brain activation regions that can simultaneously select important genetic variants. At Level 1, we focus on identifying the consistent activation regions across subjects, where the brain activation intensity may be different for different subjects. At Level 2, we are interested in identifying the important genetic variants (such as SNPs) that are strongly associated with brain activation intensities.

### 2.1. Two-level model

Suppose we collect brain images consisting of *p* voxels in a brain region ℬ ⊂ ℝ^3^ and genetic variants of *m* SNPs from *n* subjects. Let *i*(*i* = 1, …, *n*) index the subject, *j*(*j* = 1, …, *p*) index the voxels and *k*(*k* = 1, …, *m*) index the SNPs. Denote by *y*_*ij*_ the observed imaging signal at voxel **v**_*j*_ ∈ ℬ. Let *S*_*ik*_ be the genetic variant for SNP *k*.

At Level 1, we model the brain signal intensity within brain activation regions by assuming *y*_*ij*_ follow a normal mixture model:


(1) $$[y_{i}(v_{j})|\phi ,\mu_{i},\sigma_{i}^{2}]\sim \text{ N}\left[\mu_{i}\text{δ}\{\phi (v_{j})\},\sigma_{i}^{2}\right],$$

where δ(*x*) = 1 if *x* > 0 and δ(*x*) = 0 if *x* ≤ 0. The level set function ϕ(**v**):ℬ → ℝ determines the brain activation regions. For any voxel **v** in the brain, if ϕ(**v**) > 0 implying that δ{ϕ(**v**_*j*_)} = 1, then it is located in a activation region and the brain signal *y*_*ij*_ has an average activation intensity μ_*i*_. Otherwise, the voxel is located outside the brain activation regions with a mean intensity zero. The parameter $\sigma_{i}^{2}$ is the variance of the signal *y*_*ij*_ across all voxels *j* for subject *i*.

At Level 2, we link the activation intensity to the genetic variant by using a regression model


(2) $$\begin{array}{l} \mu_{i}\sim \text{N}\left(\overset{m}{\underset{k=1}{\sum }}S_{ik}\eta_{k},\tau_{\mu }^{2}\right), \end{array}$$

where η_*k*_ is the genetic effects of SNP *k* on the brain activation intensity. The variance parameter $\tau_{\mu }^{2}$ characterizes the variability of the average activation intensity that are not from the genetic variants.

### 2.2. Prior specifications

In this section, we discuss the prior specifications for models (1) and (2).

At Level 1, to guarantee the robustness and flexibility of modeling the activation regions shape, we assign a Gaussian process prior to the level set function ϕ(**v**) with mean zero and covariance kernel function, denoted as


ϕ~GP(0,κ),

where κ(**v**, **v**′):ℬ × ℬ → ℝ is a symmetric positive definite kernel function.

At Level 2, we impose sparsity on η_*k*_ to identify the important SNP sets that are strongly associated with the brain activation intensity. To achieve this goal, we adopt the spike-and-slab prior proposed by Ishwaran and Rao (2005b):


$$\begin{array}{l} \left[\eta_{k}|\gamma_{k},\tau_{k}^{2}\right]\sim \text{N}\left[0,\gamma_{k}\tau_{k}^{2}\right],\;\left[\gamma_{k}|\nu_{0},w\right]\sim \left(1-w\right)\delta_{\nu_{0}}+w\delta_{1},\\ w\sim \text{Uniform}\left[0,1\right], \end{array}$$

where δ_*v*_(·) refers to a point mass measure at a real value *v*. The parameter *w* is the prior inclusion probability indicating how likely each feature is to be selected. Pre-defined value ν_0_ usually select very small so that the “spike” (δ_ν_0__, i.e., $\text{N}\left[0,\nu_{0}\tau_{k}^{2}\right]$) part and “slab” (δ_1_, i.e., $\text{N}\left[0,\tau_{k}^{2}\right]$) part can be mostly differentiated from each other.

For all the variance parameters $\sigma_{i}^{2}$, $\tau_{\mu }^{2}$, and $\tau_{k}^{2}$, we assume they are mutually indenpendent and follow conjugate priors:


$$\sigma_{i}^{2}\sim \text{IG}\left(a_{1},a_{2}\right),\;\tau_{\mu }^{2}\sim \text{IG}\left(b_{1},b_{2}\right),\;\tau_{k}^{2}\sim \text{IG}\left(c_{1},c_{2}\right),$$

where IG(*w*_1_, *w*_2_) represents an inverse gamma prior with shape *w*_1_ and rate *w*_2_.

### 2.3. Model representation

To implement posterior computation algorithm, we need to consider model approximations. First, we consider the basis expansion approximation $\phi (v)=\sum_{l=1}^{L}\beta_{l}\psi_{l}(v)$ with $\beta_{l}\overset{\text{iid}}{\sim }\text{N}\left(0,\Lambda \right)$, where {ψ_*l*_(·)} and {λ_*l*_} are respectively eigen functions and eigenvalues for the kernel function κ(·, ·) that are shared cross all patient samples. Here, *L* is the number of basis functions and it can be determined according to the proportion of the variation of the GP, denoted as α∈{0, 1}, that can be explained by the basis expansions, i.e., $\min\left\{L:\left(\overset{L}{\underset{l=1}{\sum }}\lambda_{l}\right)\leq \overset{\infty }{\underset{l=1}{\sum }}\lambda_{l}\geq \alpha \right\}$. A common choice of α is around 0.7 to 0.8. Second, we introduce the function $H_{\epsilon }\left[x\right]=\frac{1}{2}\left[1+\frac{2}{\pi }\arctan\left(\frac{x}{\epsilon }\right)\right]$ with *H*_ϵ_[*x*] → δ[*x*] as ϵ → 0. Note that its first derivative is $H_{\epsilon }^{\left(1\right)}\left[x\right]=\frac{1}{\pi }\frac{\epsilon }{\epsilon^{2}+x^{2}}$. Let $H_{\epsilon }(\beta )={\left\{H_{\epsilon }(\psi_{1}^{\text{T}}\beta ),\cdots ,H_{\epsilon }(\psi_{p}^{\text{T}}\beta )\right\}}^{\text{T}}={\left(H_{1},\cdots ,H_{p}\right)}^{T}.$

Write $y_{i}={\left(y_{i1},\ldots ,y_{ip}\right)}^{\text{T}}$, **S** = (*S*_*ik*_), $\mu ={\left(\mu_{1},\ldots ,\mu_{n}\right)}^{\text{T}}$, $\beta ={\left(\beta_{1},\ldots ,\beta_{L}\right)}^{\text{T}}$ and $\Psi ={\left(\Psi_{1},\ldots ,\Psi_{p}\right)}^{\text{T}}$ with $\psi_{j}={\left[\psi_{1,j},\ldots ,\psi_{L,j}\right]}^{\text{T}}$ and ψ_*l, j*_ = ψ_*l*_(**v**_*j*_). Then our Bayesian hierarchical model with prior specifications can be represented as


(3) $$\begin{array}{l} y_{i}|\beta ,\mu_{i},\sigma_{i}^{2}\sim \;{\text{N}}_{p}\left[\mu_{i}\;H_{\epsilon }(\beta ),\sigma_{i}^{2}I_{p}\right],\;\beta \sim \;{\text{N}}_{L}\left[0,\Lambda_{L}\right],\\ \mu \sim \;{\text{N}}_{n}[S^{T}\eta ,\tau_{\mu }^{2}1_{n}],\\ \eta |\gamma ,\tau^{2}\sim \;{\text{N}}_{m}[0,\Gamma (\gamma ,\tau^{2})],\;\gamma_{k}|w\sim (1-w)\delta_{\nu_{0}}+w\delta_{1},\\ w\sim \text{Uniform}[0,1],\\ \sigma_{i}^{2}\sim \text{ IG}[a_{1},a_{2}],\;\tau_{\mu }^{2}\sim \text{ IG}[b_{1},b_{2}],\;\tau_{k}^{2}\sim \text{ IG}[c_{1},c_{2}], \end{array}$$

where **Γ** is a diagonal matrix with (*k, k*) element being $\gamma_{k}\tau_{k}^{2}$.

### 2.4. The model with non-sparse prior

We also consider a conjugate normal prior on **η** without imposing sparsity which leads to more efficient posterior computation. The model is represented as


$$\begin{array}{l} y_{i}|\beta ,\mu_{i},\sigma_{i}^{2}\sim \;{\text{N}}_{p}\left[\mu_{i}\;H_{\epsilon }(\beta ),\sigma_{i}^{2}I_{p}\right],\;\beta \sim \;{\text{N}}_{L}\left[0,\Lambda_{L}\right],\\ \mu \sim \;{\text{N}}_{q}[S^{T}\eta ,\tau_{\mu }^{2}I_{q}],\;\eta \sim \;{\text{N}}_{m}[0,\tau_{\eta }^{2}I_{m}],\\ \sigma_{i}^{2}\sim \text{ IG}[a_{1},a_{2}],\;\tau_{\mu }^{2}\sim \text{ IG}[b_{1},b_{2}],\;\tau_{k}^{2}\sim \text{ IG}[d_{1},d_{2}], \end{array}$$

### 2.5. Posterior computation and variable selection

For posterior computation, we adopt the Riemann Manifold Metropolis adjusted Langevin algorithm (MMALA) (Girolami and Calderhead, 2011) and Stochastic Search Variable Selection (SSVS) George and McCulloch (1997) within Gibbs sampling.

For the variable selection, we apply an *ad-hoc* method based on posterior credible intervals. For correlating the variables (clinical or SNPs) with brain image intensity levels, we use the null hypothesis that SNP *k* is uncorrelated with the intensity level inside the activation region (*H*_0_:η_*k*_ = 0) and the alternative hypothesis that SNP *k* is not uncorrelated with the intensity level inside the activation region (*H*_*a*_:η_*k*_≠0). Based on the marginal posterior distribution for η_*k*_, if 0 is included in the posterior 99% credible interval, we assign γ_*k*_ = 1, otherwise γ_*k*_ = 0 where γ_*k*_ is the same indicator variable introduced in SSVS. We approximate the posterior inclusion probability of SNP *k*: *Eγ*_*k*_ using the averaged values after burn-ins ${\bar{\gamma }}_{k}$. Then the SNPs with posterior inclusion probability larger than 0.01 are selected as important.

The details of derivations and posterior computation algorithms the Bayesian level set method with spike-and-slab prior and normal prior are provided in the Supplementary material.

## 3. Simulations

We tested the performance for learning activation region shapes and selection influential variables using proposed method starting from the simplest scenario and then gradually extended to the most complicated scenario. For the simplest simulation setting, we simulated a single subject, 2D imaging data and zero predictor matrix, i.e., set *n* = 1, *d* = 2, **S** = 0 thus no variable selection was involved. For the most complicated simulation setting, we simulated multiple subjects, 3D imaging data and utilized the predictors in real data analysis for selection.

### 3.1. Single subject with 2D image and no variable selection

In this simulation study, the objective is to test the Bayesian nonparametric level set method for random shape fitting. We simulated 2D images of size 150 × 150 on a square region [−1, 1]^2^ (*d* = 2). We considered three activation region shapes: circles, squares and random shapes. We simulated data by setting σ^2^ = 1, and the signal intensities μ and the level set function were set as follows:

- Circle shapes: set the signal intensity μ = 1 (weak) and the true level set function $\phi (v)=\exp\{-0.5(v_{1}^{2}+v_{2}^{2})\}-0.8$
- Square shapes: set the signal intensity μ = 3 (strong) and the true level set function ϕ(**v**) = exp{−0.5(|*v*_1_|+|*v*_2_|)}−0.8
- Random shapes: set the signal intensity μ = 2 (intermediate) and draw the true level set from a Gaussian process with mean zero and covariance kernel $\kappa \left(v_{1},v_{2}\right)=\exp\left(-10{\left(v_{1}-v_{2}\right)}^{2}\right)$

**Figure 1:**
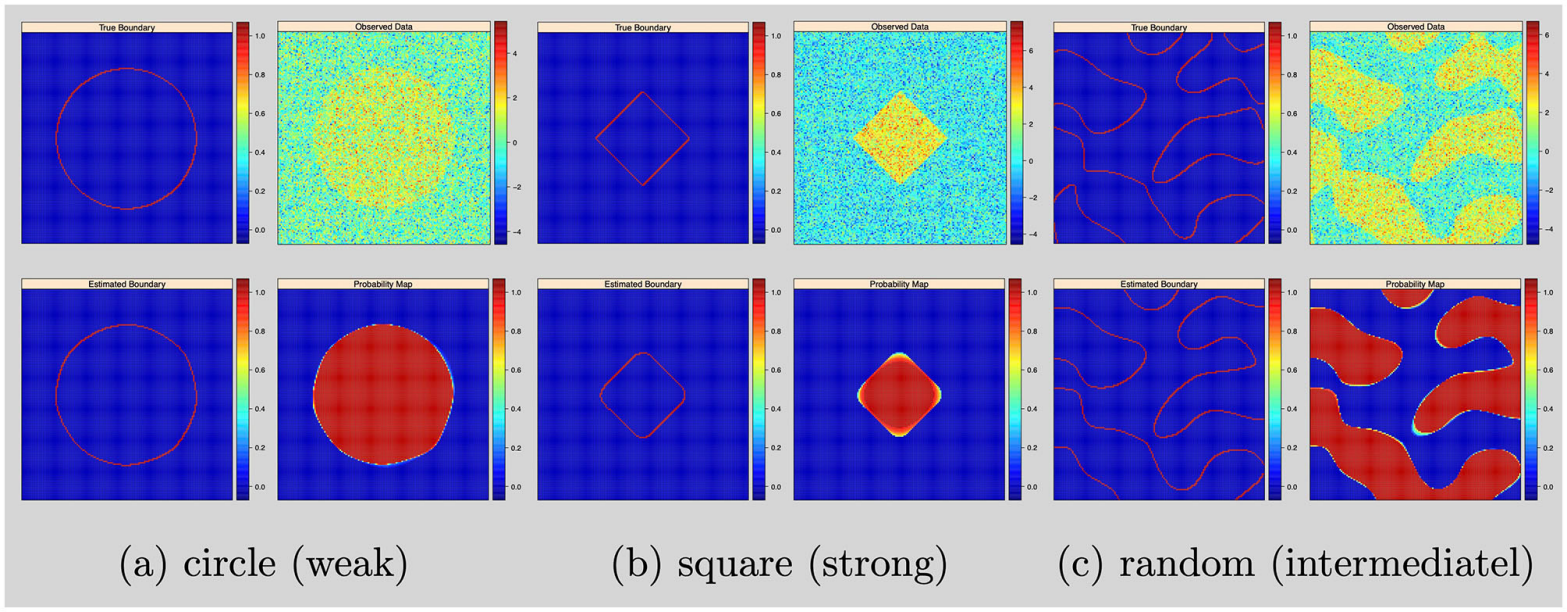
Single subject with 2D image and no variable selection: from top to bottom, left to right: simulated boundary in red, simulated intensity data, estimated boundary in red and inclusion probability map. **(a)** Circle (weak). **(b)** Square (strong). **(c)** Random (intermediatel).

For the posterior computation, we set ϵ = 1 × 10^−3^ and run 50,000 iterations with 20,000 burn-ins. The shape estimation results were presented in Figure 1.

### 3.2. Multi-subjects with 3D image and no variable selection

We evaluated the proposed method on a total of *m* = 50 subjects with 3D images simulated for each of them. The 3D image grid was of 20 × 20 × 20 (*p* = 8, 000) on a square region [−1, 1]^2^ (*d* = 3). Like simulation 3.1, we set **S** = 0 so that there is no variable selection involved. We considered three different shapes of activation region: spheres, diamonds, and random shapes. We set $\sigma_{i}^{2}=1,i=1,\ldots ,n$. The signal intensities μ_*i*_ (*i* = 1, …, *n*) and the level set function ϕ(**v**) were set as follows:

- Sphere shapes: set the signal intensity μ_*i*_~*N*(1, 1) and the true level set function $\phi (v)=\exp\{-0.5(v_{1}^{2}+v_{2}^{2}+v_{3}^{2})\}-0.7$
- Diamond shapes: set the signal intensity μ_*i*_~*N*(3, 1) and the true level set function ϕ(**v**) = exp{−0.5(|*v*_1_|+|*v*_2_|+|*v*_3_|)}−0.6
- Random signal shapes: set the signal intensity μ_*i*_~*N*(2, 1) and draw the true level set from a Gaussian process with mean zero and covariance kernel $\kappa (v_{1},v_{2})=\exp(-10{\left(v_{1}-v_{2}\right)}^{T}(v_{1}-v_{2}))$

**Figure 2:**
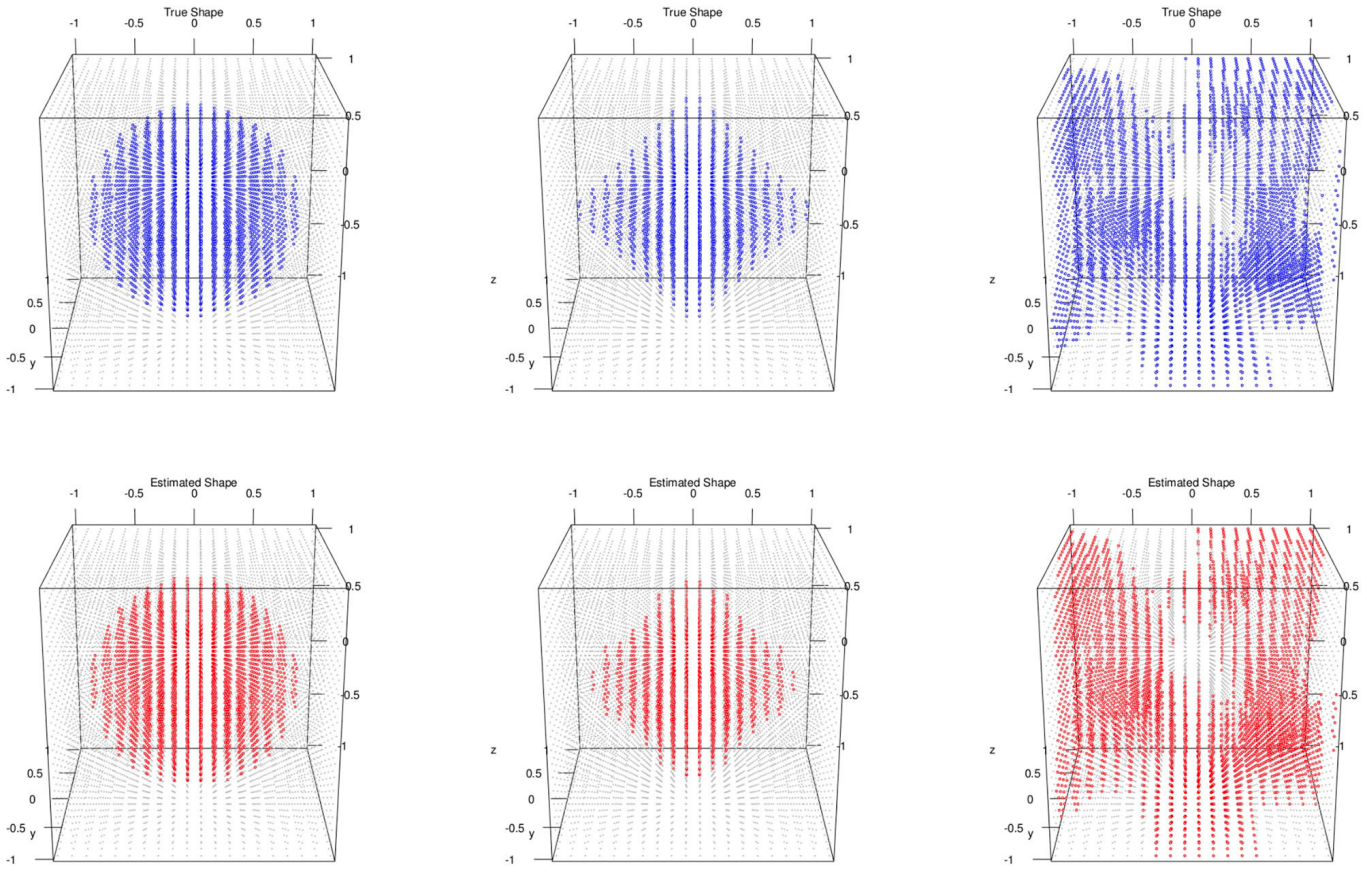
Multiple subjects with 3D image and no variable selection: **top**/**bottom**: simulated/estimated shapes; classification accuracies; **left** to **right**: MSE(**μ**) are sphere 0.98, 0.000218; diamond 0.98, 0.000728; random 0.96, 0.000158.

For the posterior computation, we set ϵ = 1 × 10^−3^, α = 0.8 as PCA percent and run 5,000 iteration with 2,000 burn-in. The shape segmentation results were respectively summarized in Figure 2.

### 3.3. Multi-subjects with 3D image and variable selection

In the simulation study, we evaluated the proposed method on the most complicated scenario where there is a total of *n* = 235 subjects with 3D images simulated for each of them. We only took the first 200 columns (*m* = 200) from the SNP matrix in real data analysis to form **S** in the simulations. We randomly selected 5 of them as signal (without loss of generosity, set η_*k*_ = 1, *k* = 1, …, 5) and the remaining 195 (η_*k*_ = 0, *k* = 6, …, 200) as noise. Like previous simulation studies, we considered three different activation region shapes with different combination of abilities for shape estimation and variable selection quantified by signal-to-noise-ratio SNR(•).


$$\begin{array}{l} \text{SNR}(\beta )=\frac{1}{n}\sum_{i=1}^{n}\text{SNR}\left(\beta |y_{i}\right)=\frac{1}{n}\sum_{i=1}^{n}\frac{\left|\mu_{i}\right|}{\sigma_{i}}\\ \text{SNR}(\eta )=\frac{\text{Var}(E\mu )}{E\epsilon^{2}}=\frac{\text{Var}(S\eta )}{E\epsilon^{2}} \end{array}$$

where SNR(**β**) is the signal-to-noise ratio for activation shape estimation and SNR(**η**) is the signal-to-noise ratio for variable selection., and in simulations, we simulated datasets of different combinations: SNR(**β**) = 8, 5, 2 and SNR(**η**) = 8, 5, 2.

For the posterior computation, we set ϵ = 1 × 10^−4^ and α = 0.75 as the proportion of variation explained in the GP approximation, which leads to 120 basis functions. We ran 6,000 iterations with 4,000 burn-ins and saved the MCMC sample for every two iterations. For each of the simulation settings, we simulated 50 datasets in total and evaluated the algorithm performance based on some proposed metrics averaged across different datasets. The voxels inside activation regions were selected if their posterior inclusion probability is larger than 0.5. The variable are selected if their posterior inclusion probability is larger than 0.02 for SSVS and 0.01 when used non-sparse prior.

For activation shape estimation and variable selection, as there are only two possible values that voxels can take: “inside the region” or “outside the region”, also two possible values that variables can take: “selected” or “not-selected”, we can summarize spatial voxels and variable selection results by their averaged accuracy, sensitivity, and specificity respectively. We also provided the averaged mean-squared-errors (MSE) for **η** and **μ**. The simulation results using SSVS are presented in Table 1 and results using non-sparse prior are presented in Table 1.

**Table 1:** Comparisons between BNLS with the spike-and-slab prior and with the normal prior in the simulation results for 3D image with multiple subjects.

**Shape**	**Sphere**			**Diamond**			**Random**		
**SNR**(β)	8			5			2		
**SNR**(**η**)	8	5	2	8	5	2	8	5	2
**BNLS with spike-and-slab prior**									
ACC (AR)	0.992	0.992	0.991	0.991	0.991	0.991	0.952	0.954	0.952
TPR (AR)	0.972	0.969	0.965	0.949	0.949	0.948	0.950	0.952	0.950
TNR (AR)	1.000	1.000	1.000	1.000	1.000	1.000	0.948	0.950	0.947
ACC (GV)	1.000	1.000	1.000	1.000	1.000	1.000	1.000	1.000	1.000
TPR (GV)	1.000	1.000	1.000	1.000	1.000	1.000	1.000	1.000	1.000
TNR (GV)	1.000	1.000	1.000	1.000	1.000	1.000	1.000	1.000	1.000
MSE(**η**)	0.000	0.000	0.000	0.000	0.000	0.000	0.000	0.000	0.000
MSE(**μ**)	0.000	0.000	0.000	0.004	0.003	0.003	0.179	0.162	0.153
**BNLS with normal prior (non-sparse prior)**									
ACC (AR)	0.991	0.991	0.991	0.993	0.992	0.992	0.954	0.952	0.954
TPR (AR)	0.969	0.969	0.969	0.956	0.950	0.955	0.952	0.950	0.954
TNR (AR)	1.000	1.000	1.000	1.000	1.000	1.000	0.952	0.951	0.948
ACC (GV)	0.926	0.909	0.894	0.926	0.907	0.898	0.931	0.923	0.893
TPR (GV)	1.000	0.970	0.865	0.990	0.970	0.850	1.000	0.945	0.840
TNR (GV)	0.924	0.907	0.895	0.924	0.906	0.900	0.929	0.923	0.894
MSE(**η**)	0.028	0.044	0.089	0.028	0.041	0.091	0.025	0.039	0.081
MSE(**μ**)	0.001	0.000	0.000	0.011	0.007	0.003	0.236	0.219	0.141

The results include parameter estimation mean squared error (MSE) and selection accuracy (ACC), true positive rate (TPR) and true negative rate (TNR) for brain activation region (AR) and genetic variants (GV) for three shapes with combinations of the signal-to-noise ratios.

The simulation studies indicate our proposed method is accurate for voxels classification and variable selection. For simulations using SSVS, even with the worse scenario when SNR(**β**) = 2 and SNR(**η**) = 2, the averaged accuracy, sensitivity and specificity for voxels classification are all above 0.94 and for variable selection are all 100%. As SNR(**β**) increased to 5 and 8, classification performance improves as expected while *SNR*(**η**) increased to 5 and 8, variable selection are all 100% accurate. For MSE of **η** and **μ**, it decreases in the general trend when SNR(**β**) increases.

Compared to Bayesian level set method with non-sparse prior for variable selection, the voxels classification is robust but the variable selection generates worse performance. If we compare the scenario when SNR(**β**) = SNR(**η**) = 2, the accuracy, sensitivity (true positive rate) and specificity (true negative rate) decrease to 0.893, 0.840, 0.894 and MSE for **η** and **μ** increases to 0.081 and 0.141. The proposed method does suffer a decrease performance as expected, but in general, the results are acceptable. We recommend applying the fast algorithm when there is exceedingly large number of candidate SNPs in the study for fast computation purpose.

To assess our model's performance relative to existing methods like FVGWAS and FGWAS, we specifically examine signal-to-noise ratios (SNR) for both brain activation region selection and genetic effects, using estimations from the analysis of ADNI data. The estimated SNR(β)≈0.5, while for η, the SNR varies between 0.5 and 1.0; hence, we consider two scenarios: SNR(η) = 0.5, 1.0. We simulate the activation region through Gaussian Processes (GP), where the spatial correlation is approximated to be 0.9 for neighboring voxels, based on PET image analysis. Given that the underlying assumptions of our Bayesian model differ from those of FVGWAS and FGWAS, the parameter estimations are not directly comparable. Therefore, we focus our comparison on activation region and SNP selection results, omitting the mean squared error (MSE) metrics. The findings are summarized in Table 2.

**Table 2:** Comparisons between FVGWAS, FGWAS, BNLS in the simulations for 3D image with multiple subjects, where the data were simulated according to the signal to noise ratios estimated from the real PET images in the ADNI study.

**Method**	**FVGWAS**		**FGWAS**		**Bayesian**	
SNR(**η**)	0.5	1.0	0.5	1.0	0.5	1.0
ACC (AR)	0.562	0.570	0.658	0.713	0.945	0.944
TPR (AR)	0.117	0.145	1.000	1.000	0.938	0.957
TNR (AR)	1.000	1.000	0.235	0.499	0.951	0.929
ACC (GV)	0.831	0.802	0.499	0.504	0.985	0.995
TPR (GV)	0.904	0.960	0.400	0.400	0.400	0.800
TNR (GV)	0.829	0.798	0.502	0.507	1.000	1.000

The results include parameter estimation mean squared error (MSE) and selection accuracy (ACC), true positive rate (TPR) and true negative rate (TNR) for brain activation region (AR) and genetic variants (GV) for two levels the signal-to-noise ratios genetic variants.

The comparative analysis indicates that FVGWAS exhibits low power in detecting activation regions but performs exceptionally well in identifying genetic variants, albeit with a slightly inflated false-positive rate. On the other hand, FGWAS, which incorporates spatial smoothness into its estimations, demonstrates high power in detecting activation regions but struggles to control the false-positive rate effectively, resulting in low specificity. In contrast, our proposed Bayesian level set method, augmented with a spike-and-slab prior, outperforms both methods in terms of activation region and genetic variant selection. Importantly, it also effectively controls the false-positive rate.

## 4. Analysis of ADNI data

We applied the proposed method on an imaging genetics study to detect genotypes that are associated with imaging phenotypes (both imaging intensities and activation shapes) in application to the Alzheimer's disease. To be specific, the primary goal was to determinate any specific gene markers that are correlated with regional activation levels in the brain, which can serve as potential indicators of disease with different levels of progression.

The data was made available by the Alzheimer's Disease Neuroimaging Initiative (ADNI). There are three different groups of subjects: 69 normal subjects (NORM), 117 mild cognitive impairment subjects (MCI), and 49 Alzheimer's disease patients (AD). In total, 235 subjects were included in the study. For genotypes, we selected 614 SNPs that are associated with 34 genes known to be potentially related to AD from the literature. In addition to the genetic data, we also included 5 clinical factors. They are: the subjects age, gender, body weight, neuropsychiatric inventory score (NPISCORE), and functional activity questionnaire score (FAQSCORE). There are 2 missing values in NPISCORE and 4 missing values in FAQSCORE. All the missing values were imputed by the mean values of observed data for the corresponding variables. We standardized each variable so that they had an average value of 0 and a variance of 1. We included 42 brain regions in the analysis. There are 12 regions located in Frontal lobe including (Frontal_Sup_L, etc.); 8 in Parietal lobe including Parietal_Sup_L, etc.; 6 in Occipital lobe including Occipital_Sup_L, etc. and 16 in Temporal lobe including Temporal_Sup_R, Hippocampus_L, etc. (L: left hemisphere, R: right hemisphere). We studied each of them at three different time points: baseline (bl), month 6 (m6), and month 12 (m12).

We applied the proposed level set image segmentation for activation region fitting, and utilized the spike and slab prior in variable selection as we only included limited number of SNPs in the study. We applied our method to each of the brain anatomical regions. The objective was to learn the brain activation region changes over time, and to select significant SNP biomarkers that were associated with the activation intensities. There were some assumptions in the model in the way we implemented. First, we borrowed the anatomical structure information by assuming separate activation regions (two anatomical regions *A* and *B*, **β**s are different: **β**(*A*)≠**β**(*B*)), independent intensity levels within subject (μ_*i*_(*A*)≠μ_*i*_(*B*)) and across subjects (μ_*i*_(*A*)≠μ_*j*_(*B*)), individual set of influential SNPs (**η**(*A*)≠**η**(*B*)). Second, we simplified our model by assuming the same level of activation within one anatomical brain region due to the fact that anatomical regions are usually small areas in the brain.

**Figure 3:**
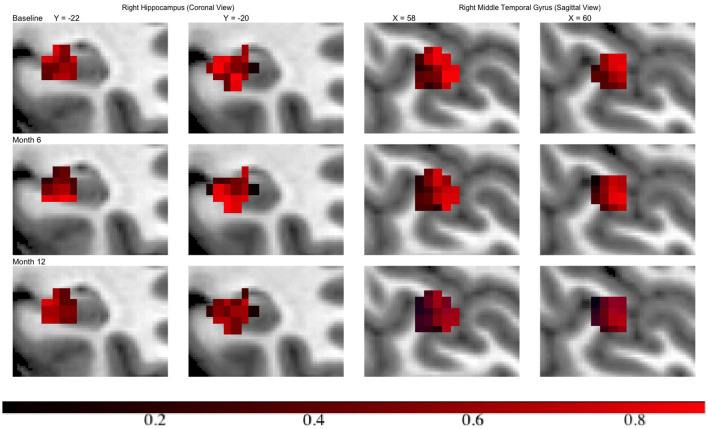
Inclusion probability map indicating changes of brain activation shapes. **Left**/**right**: the hippocampus from the right hemisphere from coronal panel/ the middle temporal gyrus from the right hemisphere at saggital panel. Points: light blue, anatomical regions; colored, activation regions. Red to dark blue: inclusion probability decreases from 1 to 0.

Across all regions in brain, the number of voxels ranges from 335 to 5104, with an average of 2134. We set ϵ = 1 × 10^−4^, α = 0.75 leading to 120 basis functions for fitting the GP, i.e., *L* = 120. Then we run the MMALA for 6,000 iterations with 4,000 burn-in and save the MCMC samples for every two iterations. The activation regions are uniquely defined by the voxels selected inside the activation. In simulation studies, voxels are selected as inside the activation regions if their posterior inclusion probabilities after burn-ins are larger (i.e., voxel **v**_*j*_ are selected if ${\bar{z}}_{j}\geq 0.5$). Compared to this hard-thresholding likewise method, in real data analysis, we combined all voxels selected across all time points together and defined as our “ROI” and then presented results in a probability map where we presented each voxel's marginal posterior inclusion probability. For instance, the progression in hippocampus region from the right hemisphere, in the middle temporal gyrus from the right hemisphere at different time points are presented in Figure 3. We observed that in both regions activations are decreased over the time along with disease progression.

**Figure 4:**
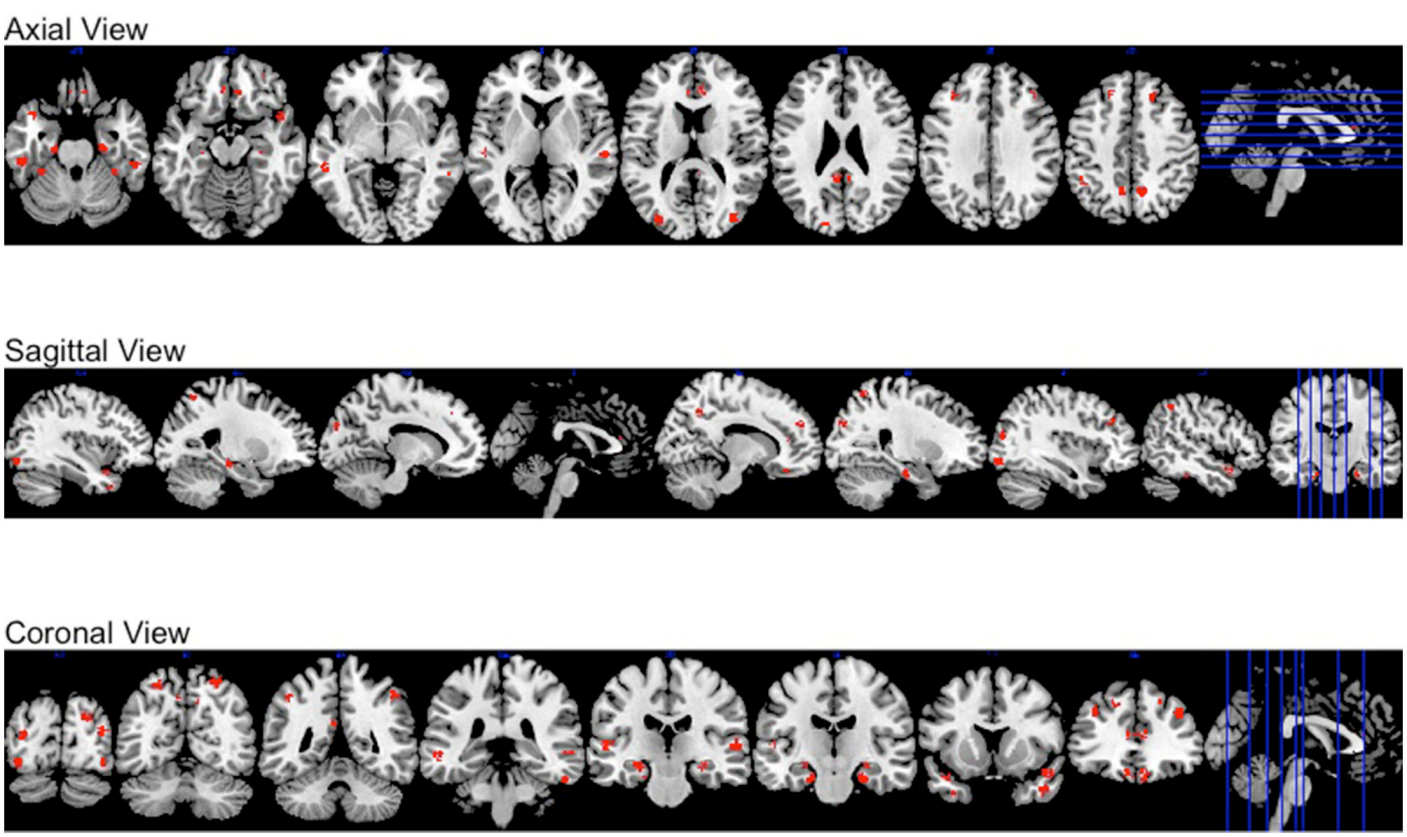
Different views of brain-wide activation regions. Red: activation regions; light blue: anatomical regions.

Moreover, all activation regions at a brain-wide level are presented at the axial, sagittal and coronal panel in Figure 4. We observed that the activations follow the human brain structure symmetry. We also presented anatomically regional activation selection table using hard-thresholding method in the Table 3.

**Table 3:** Anatomical region-wise results: number of voxels inside activation regions at different time points using hard-thresholding criterion, time points for each selected SNPs within one region.

**Region name**	**nvoxels at bl**	**nvoxels at m6**	**nvoxels at m12**	**rs16940638 (ADAM10)**	**rs10512186 (DAPK1)**	**rs677066 (CR1)**	**rs3734404 (NEDD9)**	**rs2274976 (MTHFR)**
Frontal_Sup_L	31	37	30	bl,m6,m12,				
Frontal_Sup_R	31	31	31	bl,m6,m12,				
Frontal_Mid_L	42	34	34	bl,	m12,	m6,m12,		
Frontal_Mid_R	32	36	37	bl,m6,m12,				
Frontal_Sup_Medial_L	41	43	41	bl,m6,				
Frontal_Sup_Medial_R	33	35	38	bl,m6,m12,				
Frontal_Mid_Orb_L	31	32	36	bl,m6,m12,				
Frontal_Mid_Orb_R	31	32	32	bl,m6,m12,				
Rectus_L	35	35	35	bl,m6,m12,	m12,			
Rectus_R	45	39	44	bl,m6,m12,	m6,m12,			
Cingulum_Ant_L	19	19	19	bl,m6,m12,				
Cingulum_Ant_R	30	30	30	bl,m6,m12,				
ParaHippocampal_L	30	30	31	bl,				
ParaHippocampal_R	30	29	31	bl,m6,m12,				
Parietal_Sup_L	42	46	42	bl,m6,m12,				
Parietal_Sup_R	47	45	47		m12,		m6,	bl,
Parietal_Inf_L	33	33	37			bl,m6,m12,		
Parietal_Inf_R	36	36	36	bl,m6,m12,				
Precuneus_L	43	45	43	bl,m6,m12,	m6,m12,			
Precuneus_R	48	47	50	bl,m6,m12,	m12,			
Cingulum_Post_L	30	30	30	bl,m6,m12,				
Cingulum_Post_R	19	19	19	bl,m6,m12,				
Temporal_Inf_L	47	47	48	bl,m6,	m6,m12,			
Temporal_Inf_R	35	35	35	bl,m6,m12,	m6,m12,			
Fusiform_L	41	42	40	bl,m6,m12,	m6,m12,			
Fusiform_R	29	29	29	bl,m6,m12,	m6,m12,			
Occipital_Sup_L	32	35	32	bl,m6,m12,	m12,			
Occipital_Mid_R	48	46	48	bl,m12,	m6,m12,			
Occipital_Inf_L	40	43	40	bl,m6,m12,	m6,m12,			
Occipital_Inf_R	44	42	45	bl,m6,m12,	m6,m12,			
Temporal_Pole_Mid_L	35	34	33	bl,m6,m12,	m12,			
Temporal_Pole_Mid_R	44	42	43	bl,m6,m12,	m6,m12,			
Temporal_Pole_Sup_L	36	36	36					
Temporal_Pole_Sup_R	41	41	41	bl,m6,m12,	m6,			
Temporal_Mid_L	42	41	46	bl,m12,	m6,m12,			
Temporal_Mid_R	36	38	33	bl,	m6,m12,			
Hippocampus_L	19	38	38	bl,m6,m12,	m6,m12,			
Hippocampus_R	31	31	27	bl,m6,m12,	m6,			
Temporal_Sup_L	39	40	40		m6,m12,	m12,		
Temporal_Sup_R	49	49	48	bl,m6,m12,	m6,m12,			
Occipital_Sup_R	36	36	36	bl,m6,m12,			m6,	
Occipital_Mid_L	46	45	51	bl,m6,m12,				

For variable selection, different from simulations studies where we selected variables based on their posterior inclusion probabilities (≥0.02), this time, we applied a more stringent rule where only SNPs with posterior inclusion probability larger than 0.5 are selected as highly-influential SNP of interest. We pooled the SNPs selected from at least one anatomical region together. As shown in the Venn diagram (Figure 5), in total there were 5 SNPs selected: SNP rs677066 (gene CR1) and SNP rs16940638 (gene ADAM10) are selected at all time points which indicated their consistent impact on the activation intensities; SNP rs2274976 (gene MTHFR) and SNP rs3734404 (gene NEDD9) were only selected at baseline and month 6 respectively; SNP rs10512186 (gene DAPK1) was selected at both month 6 and month 12. For each of the selected SNP, the number of regions related at different time points, region names, and region-related lobe names can be found in Table 4.

**Figure 5:**
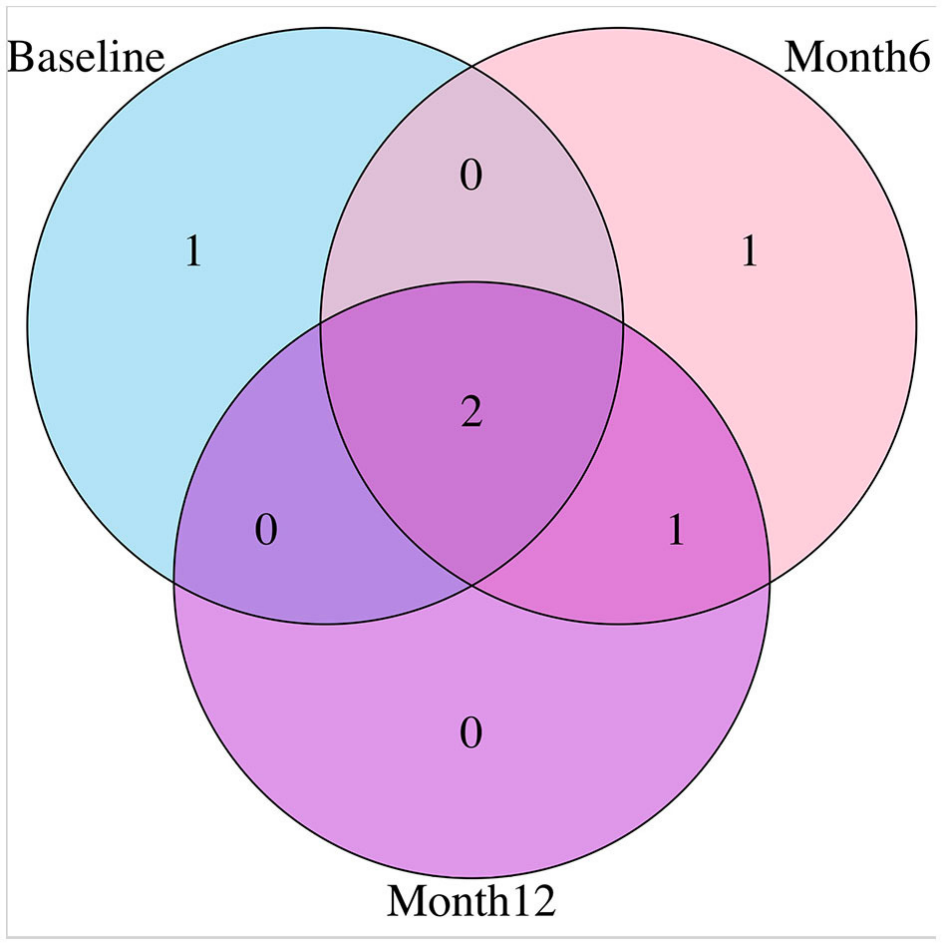
Venn diagram presenting relations of SNPs selected at different time points.

**Table 4:** Selected SNPs with related anatomical regions at different time points.

**SNP**	**#regions**	**Regions at baseline**	**Regions at month 6**	**Regions at month 12**	**Lobes**
rs16940638 Gene ADAM10	bl: 38 m6: 33 m12: 33	Cingulum_Ant_L Cingulum_Ant_R Cingulum_Post_L Cingulum_Post_R Frontal_Mid_L Frontal_Mid_Orb_L Frontal_Mid_Orb_R Frontal_Mid_R Frontal_Sup_L Frontal_Sup_Medial_L Frontal_Sup_Medial_R Frontal_Sup_R Fusiform_L Fusiform_R Hippocampus_L Hippocampus_R Occipital_Inf_L Occipital_Inf_R Occipital_Mid_L Occipital_Mid_R Occipital_Sup_L Occipital_Sup_R ParaHippocampal_L ParaHippocampal_R Parietal_Inf_R Parietal_Sup_L Precuneus_L Precuneus_R Rectus_L Rectus_R Temporal_Inf_L Temporal_Inf_R Temporal_Mid_L Temporal_Mid_R Temporal_Pole_Mid_L Temporal_Pole_Mid_R Temporal_Pole_Sup_R Temporal_Sup_R	Cingulum_Ant_L Cingulum_Ant_R Cingulum_Post_L Cingulum_Post_R Frontal_Mid_Orb_L Frontal_Mid_Orb_R Frontal_Mid_R Frontal_Sup_L Frontal_Sup_Medial_L Frontal_Sup_Medial_R Frontal_Sup_R Fusiform_L Fusiform_R Hippocampus_L Hippocampus_R Occipital_Inf_L Occipital_Inf_R Occipital_Mid_L Occipital_Sup_L Occipital_Sup_R ParaHippocampal_R Parietal_Inf_R Parietal_Sup_L Precuneus_L Precuneus_R Rectus_L Rectus_R Temporal_Inf_L Temporal_Inf_R Temporal_Pole_Mid_L Temporal_Pole_Mid_R Temporal_Pole_Sup_R Temporal_Sup_R	Cingulum_Ant_L Cingulum_Ant_R Cingulum_Post_L Cingulum_Post_R Frontal_Mid_Orb_L Frontal_Mid_Orb_R Frontal_Mid_R Frontal_Sup_L Frontal_Sup_Medial_R Frontal_Sup_R Fusiform_L Fusiform_R Hippocampus_L Hippocampus_R Occipital_Inf_L Occipital_Inf_R Occipital_Mid_L Occipital_Mid_R Occipital_Sup_L Occipital_Sup_R ParaHippocampal_R Parietal_Inf_R Parietal_Sup_L Precuneus_L Precuneus_R Rectus_L Rectus_R Temporal_Inf_R Temporal_Mid_L Temporal_Pole_Mid_L Temporal_Pole_Mid_R Temporal_Pole_Sup_R Temporal_Sup_R	Frontal occipital parietal temporal
rs10512186 Gene DAPK1	bl: 0 m6: 17 m12: 21		Fusiform_L Fusiform_R Hippocampus_L Hippocampus_R Occipital_Inf_L Occipital_Inf_R Occipital_Mid_R Precuneus_L Rectus_R Temporal_Inf_L Temporal_Inf_R Temporal_Mid_L Temporal_Mid_R Temporal_Pole_Mid_R Temporal_Pole_Sup_R Temporal_Sup_L Temporal_Sup_R	Frontal_Mid_L Fusiform_L Fusiform_R Hippocampus_L Occipital_Inf_L Occipital_Inf_R Occipital_Mid_R Occipital_Sup_L Parietal_Sup_R Precuneus_L Precuneus_R Rectus_L Rectus_R Temporal_Inf_L Temporal_Inf_R Temporal_Mid_L Temporal_Mid_R Temporal_Pole_Mid_L Temporal_Pole_Mid_R Temporal_Sup_L Temporal_Sup_R	Frontal occipital parietal temporal
rs677066 Gene CR1	bl: 1 m6: 2 m12: 3	Parietal_Inf_L	Frontal_Mid_L Parietal_Inf_L	Frontal_Mid_L Parietal_Inf_L Temporal_Sup_L	Frontal parietal temporal
rs3734404 Gene NEDD9	bl: 0 m6: 2 m12: 0		Occipital_Sup_R Parietal_Sup_R		Parietal temporal
rs2274976 Gene MTHFR	bl: 1 m6: 0 m12: 0	Parietal_Sup_R			Parietal

Based on SNP-activation relation, we observe that ADAM10 (SNP rs16940638) is universally associated with the majority of the anatomical brain regions across time: 38 out of 42 at baseline, 33 out of 42 at month 6, and 33 out of 42 at month 12. The SNP has been identified as one of the significant genetic variants using genome-wide association studies (GWAS) and mediation analyses where the objective was to detect SNPs that influence psychiatric and cognitive traits through intermediaries, and would not be detected otherwise (Bi et al., 2017). The biological function of the gene, ADAM10, is to proteolytic release cell-surface proteins, including TNF-alpha, heparin-binding epidermal growth-like factor, Notch receptors, and amyloid precursor protein (APP) in the non-amyloidogenic manner (Rabquer et al., 2010; Jouannet et al., 2016; Seegar et al., 2017). The regulatory role of ADAM10 in the brain has been well-documented (Saftig and Lichtenthaler, 2015).

In our results, rs677066 is associated with inferior parietal left in all three time points, while it is associated with middle frontal gyrus left at both 6 months and 12 months, but not at baseline., and it is associated with superior temporal gyrus left only at month 12. In existing literature, rs677066 (gene: CR1) is among the top SNPs to be related with AD gene pathway implicated in Alzheimer's disease (Silver et al., 2012). It was also found to play a role in the spontaneous idiopathic preterm birth (McElroy, 2013). The CR1 gene encodes a type I membrane glycoprotein that typically mediates cellular binding with immune complexes (Schifferli et al., 1988). The exact molecular mechanism of CR1's involvement with Alzheimer's is yet to be elucidated.

The role of DAPK1 (SNP rs10512186) and NEDD9 (SNP rs3734404) in the brain are not documented based on existing literature, but their functionalities can be inferred. DAPK1 (rs10512186) may be related to the late-onset of Alzheimer's disease as it is only selected at month 6 and month 12. Functionally, DAPK1 is a death-associated protein kinase that mediates a number of cellular processes including apoptosis and autophagy (Singh et al., 2016). The genetic variations in DAPK1 are known to be related with late-onset of Alzheimer's disease (Li et al., 2006).

NEDD9 (neural precursor cell expressed developmentally down-regulated protein 9) plays a key role in tyrosine-kinase signaling related to cell adhesion (Regelmann et al., 2006). The role of rs3734404/NEDD9 in AD is incomplete and inconsistent: some literature argued its functionalities with late-onset Alzheimer's disease (Strittmatter et al., 1993; Li et al., 2007) while some literature reported no association between the SNP genotype and AD (Chapuis et al., 2008). In our results, NEDD9 is associated with two regions only at month 6—superior occipital gyrus right and superior parietal gyrus right, indicating a transient role of the gene in disease development.

In our results, the MTHFR rs2274976 polymorphism is associated with superior parietal gyrus right at baseline. The presence of another MTHFR polymophism, rs180113 is associated with increased risk for Alzheimer's disease, adult depression, and neural tube defects in the fetus, *etc* (Trimmer, 2013). The gene MTHFR codes the protein methylenetetrahydrofolate reductase, which catalyzes a reaction involving the vitamin folate, and also plays a role processing amino acids (Wan et al., 2018). The level of serum folate is lower in AD patients, and folate deficiency is associated with higher risk of AD (Zhang et al., 2021; Prado et al., 2023). Our results indicates the association may be more critical at the onset of the disease. In addition, the variant of rs2274976 in MTHFR results in an arginine-to-glutamic acid change at amino acid 594. But as it is less frequent, its functionality is largely unknown. It may need additional attention based on our result.

## 5. Conclusion and discussion

We have developed a novel Bayesian hierarchical model in imaging genetics studies for simultaneous activation shape estimation and variable selection. Our approach can jointly estimate the brain activation regions after accounting for external sources of clinical factors and genetic variation. To the best of our knowledge, currently there is no method that shares the same goal with us. We also borrow the anatomical brain segmentation as prior information. Our approach can detect important genetic and demographic factors associated with activation intensities inside activation regions. We applied the new method to an ADNI dataset as real data application. The method yielded new results that are interpretable, and pointed to some important loci that deserve further biological investigations.

On the other hand, our method does suffer from some limitations. First, our method uses the assumption that all averaged intensities inside are shared across all activation regions as long as they are anatomically the same, which is a relatively strong assumption. Mathematically speaking, the μ_*i*_ can be further extended to an activation-region-specific variable: μ_*i, r*_, where *r* can be pre-specified by some spatial clustering methods. Second, the proposed method is limited by computational speed. It should be optimized so that it can be scalable to larger number of SNPs which is common to GWAS studies.

The study also has some limitations in its real data application. In this study, we limited our analysis to 614 SNPs associated with 34 genes known to be potentially related to AD. The purpose is to study which genotypes among the selection had an effect at the brain image level during the first year of the onset the disease. Thus genes whose relation with the disease manifests in later stages will be not identified in this analysis. As an example, the Apolipoprotein E (APOE) gene is a well-known risk factor due to its influence on blood cholesterol. Although it was included in our analysis, APOE was not found to be significantly associated with brain activation in the FDG-PET data, presumably because APOE variants act in a more global manner, and are not directly linked to the activation level of brain regions in the specific data type.

In this work, the genetic data used only involved genotyping data. Although genetic variations can shed some light on the potential association between genes and brain region activation during AD development, it cannot elucidate detailed molecular mechanisms. In future works, we will try to include gene expression and other data types to further study the mechanisms behind the genetic associations.

## Data availability statement

Publicly available datasets were analyzed in this study. This data can be found at: https://adni.loni.usc.edu.

## Author contributions

JK and TY contributed to conception and design of the study. ZJ implemented the algorithm, performed simulation studies and real data analysis, and wrote the first draft of the manuscript. All authors formulated the statistical model, derived posterior computation algorithm, contributed to manuscript revision, read, and approved the submitted version.
