# Supplementary material for: Bayesian nonparametric method for genetic dissection of brain activation region
Source: Front Neurosci. 2023 Oct 18;17:1235321. doi: 10.3389/fnins.2023.1235321 (PMC10618557; doi:10.3389/fnins.2023.1235321)
Supplement: Supplementary file 1 [file Data_Sheet_1.pdf]

Supplementary Material for “Bayesian nonparametric method for genetic dissection of brain activation region” by Jin, Kang and Yu

1 DERIVATION OF POSTERIOR COMPUTATION

1.1 Bayesian Level Set Methods with Spike-and-Slab Prior

Let $\mathbf{Y} = (\mathbf{y}_1, \ldots, \mathbf{y}_n)^{\mathrm{T}}$ be the signal matrix. Let $\boldsymbol{\sigma}^2 = (\sigma_1^2, \ldots, \sigma_n^2)$ and $\boldsymbol{\tau}^2 = (\tau_1^2, \ldots, \tau_m^2)$. The joint posterior distribution is given by

$$
\begin{aligned}
&\pi(\boldsymbol{\beta}, \boldsymbol{\mu}, \boldsymbol{\sigma}^2, \boldsymbol{\eta}, \boldsymbol{\gamma}, \boldsymbol{\tau}^2, \tau_\nu^2, w \mid \mathbf{Y}) \\
&\propto \pi(\mathbf{Y}|\boldsymbol{\beta}, \boldsymbol{\mu}, \boldsymbol{\sigma}^2)\pi(\boldsymbol{\beta})\pi(\boldsymbol{\sigma}^2)\pi(\boldsymbol{\mu}|\boldsymbol{\eta}, \tau^2)\pi(\boldsymbol{\eta}|\boldsymbol{\gamma}, \boldsymbol{\tau}^2)\pi(\boldsymbol{\gamma}|w)\pi(\boldsymbol{\tau}^2)\pi(\tau_\mu^2)\pi(w)
\end{aligned}
$$

The full conditional posterior distributions of all the parameters in the Metropolis-Hasting (RMMALA) within Gibbs sampling are derived as follows.

First, we derive the RMMALA algorithm to update $\boldsymbol{\beta}$ given all other parameters. The log full conditional density of $\boldsymbol{\beta}$ is given by

$$
\begin{aligned}
\pi(\boldsymbol{\beta}|\bullet) &\propto \pi(\mathbf{Y}|\boldsymbol{\beta}, \boldsymbol{\mu}, \boldsymbol{\sigma}^2)\pi(\boldsymbol{\beta}) \\
&\propto \exp\left\{-\frac{1}{2}\left[\sum_{i=1}^{q}\frac{1}{\sigma_i^2}(\mathbf{y}_i - \mu_i\mathbf{H}_\epsilon(\boldsymbol{\beta}))^T(\mathbf{y}_i - \mu_i\mathbf{H}_\epsilon(\boldsymbol{\beta})) + \boldsymbol{\beta}^T\boldsymbol{\Lambda}^{-1}\boldsymbol{\beta}\right]\right\}
\end{aligned}
$$

Then log full conditional distribution of $\boldsymbol{\beta}$ is given by

$$
\mathcal{L}(\boldsymbol{\beta}) = \log[\pi(\boldsymbol{\beta} \mid \bullet)] = C - \frac{1}{2}\left[\sum_{i=1}^{q}\frac{1}{\sigma_i^2}\sum_{j=1}^{p}\left(y_{ij} - \mu_i H_\epsilon\left[\sum_{l=1}^{L}\beta_l\psi_{l,j}\right]\right)^2 + \sum_{l=1}^{L}\frac{\beta_l^2}{\lambda_l}\right],
$$

and

$$
\begin{aligned}
\frac{\partial\mathcal{L}}{\partial\beta_l} &= \sum_{i=1}^{q}\sum_{j=1}^{p}\frac{\mu_i}{\sigma_i^2}\psi_{l,j}H_\epsilon^{(1)}\left[\boldsymbol{\psi}_j^{\mathrm{T}}\boldsymbol{\beta}\right]\left(y_{ij} - \mu_i H_\epsilon\left[\boldsymbol{\psi}_j^{\mathrm{T}}\boldsymbol{\beta}\right]\right) - \frac{\beta_l}{\lambda_l} \\
\frac{\partial^2\mathcal{L}}{\partial\beta_l^2} &= \sum_{i=1}^{q}\sum_{j=1}^{p}\left\{\frac{\mu_i}{\sigma_i^2}\psi_{l,j}^2H_\epsilon^{(2)}\left[\boldsymbol{\psi}_j^{\mathrm{T}}\boldsymbol{\beta}\right]\left(y_{ij} - \mu_i H_\epsilon\left[\boldsymbol{\psi}_j^{\mathrm{T}}\boldsymbol{\beta}\right]\right) - \frac{\mu_i^2}{\sigma_i^2}\psi_{l,j}^2H_\epsilon^{2(1)}\left[\boldsymbol{\psi}_j^{\mathrm{T}}\boldsymbol{\beta}\right]\right\} - \frac{1}{\lambda_l} \\
\frac{\partial^2\mathcal{L}}{\partial\beta_l\partial\beta_k} &= \sum_{i=1}^{q}\sum_{j=1}^{p}\left\{\frac{\mu_i}{\sigma_i^2}\psi_{l,j}\psi_{k,j}\mu H_\epsilon^{(2)}\left[\boldsymbol{\psi}_j^{\mathrm{T}}\boldsymbol{\beta}\right]\left(y_{ij} - \mu_i H_\epsilon\left[\boldsymbol{\psi}_j^{\mathrm{T}}\boldsymbol{\beta}\right]\right) - \frac{\mu_i^2}{\sigma_i^2}\psi_{l,j}\psi_{k,j}H_\epsilon^{2(1)}\left[\boldsymbol{\psi}_j^{\mathrm{T}}\boldsymbol{\beta}\right]\right\}, \quad l \neq k
\end{aligned}
$$

This further implies that

$$\begin{aligned}
\nabla_{\boldsymbol{\beta}}\mathcal{L}(\boldsymbol{\beta}) &= \sum_{i=1}^{q}\frac{\mu_i}{\sigma_i^2}\sum_{j=1}^{p}(y_{ij}-\mu_i H_j)H_j^{(1)}\boldsymbol{\psi}_j - \boldsymbol{\Lambda}^{-1}\boldsymbol{\beta} \\
&= \left(\frac{\boldsymbol{\mu}}{\boldsymbol{\sigma}^2}\right)^T\left(Y_{q\times p}-\boldsymbol{\mu}_{q\times 1}H_\epsilon^T\left[\boldsymbol{\psi}_j^{\mathrm{T}}\boldsymbol{\beta}\right]_{1\times p}\right) diag(H_{p\times 1}^{(1)})_{p\times p}\boldsymbol{\psi}_j - \boldsymbol{\Lambda}^{-1}\boldsymbol{\beta}, \\
\mathbf{G}(\boldsymbol{\beta}) &= \{g_{l,k}(\boldsymbol{\beta})\}_{L\times L},
\end{aligned}$$

where

$$\begin{aligned}
g_{l,l}(\boldsymbol{\beta}) &= -\mathrm{E}[\frac{\partial^2 g}{\partial\beta_l^2}] = \sum_{i=1}^{q}\sum_{j=1}^{p}\frac{\mu_i^2}{\sigma_i^2}\psi_{l,j}^2 H_\epsilon^{2(1)}\left[\boldsymbol{\psi}_j^{\mathrm{T}}\boldsymbol{\beta}\right] + \frac{1}{\lambda_l} \\
g_{l,k}(\boldsymbol{\beta}) &= -\mathrm{E}[\frac{\partial^2 g}{\partial\beta_l\partial\beta_k}] = \sum_{i=1}^{q}\sum_{j=1}^{p}\frac{\mu_i^2}{\sigma_i^2}\psi_{l,j}\psi_{k,j} H_\epsilon^{2(1)}\left[\boldsymbol{\psi}_j^{\mathrm{T}}\boldsymbol{\beta}\right].
\end{aligned}$$

Thus, the proposal distribution for $\boldsymbol{\beta}$ of the RMMALA is given by

$$\boldsymbol{\beta}^* \sim \mathrm{N}\left[\boldsymbol{\beta}+\frac{\Delta^2}{2}\mathbf{G}^{-1}(\boldsymbol{\beta})\nabla_{\boldsymbol{\beta}}\mathcal{L}(\boldsymbol{\beta}), \Delta^2\mathbf{G}^{-1}(\boldsymbol{\beta})\right],$$

Next, we derive the Gibbs sampler to update all other algorithms. The full conditional of $\boldsymbol{\mu}$ is given by

$$\begin{aligned}
\pi(\boldsymbol{\mu}\mid\bullet) &\propto \pi(\mathbf{Y}|\boldsymbol{\beta},\boldsymbol{\mu},\boldsymbol{\sigma}^2)\pi(\boldsymbol{\mu}|\boldsymbol{\eta},\tau_\mu^2) \\
&\propto \exp\left\{-\frac{1}{2}\left[\sum_{i=1}^{q}\frac{1}{\sigma_i^2}(\mathbf{y}_i-\mu_i\mathbf{H}_\epsilon(\boldsymbol{\beta}))^T(\mathbf{y}_i-\mu_i\mathbf{H}_\epsilon(\boldsymbol{\beta}))+\sum_{i=1}^{q}\frac{1}{\tau_\mu^2}(\mu_i-\mathbf{S}_i\boldsymbol{\eta})^2\right]\right\} \\
&\propto \exp\left\{-\frac{1}{2}\left[\sum_{i=1}^{q}\left(\frac{1}{\sigma_i^2}\sum_{j=1}^{p}H_j^2+\frac{1}{\tau_\mu^2}\right)\mu_i^2 - 2\left(\frac{1}{\sigma_i^2}\sum_{j=1}^{p}H_j y_{ij}+\frac{1}{\tau_\mu^2}\sum_{k=1}^{m}S_{ik}\eta_k\right)\mu_i\right]\right\}
\end{aligned}$$

This implies that we can update each μ_i by sampling from

$$[\mu_i\mid\bullet] \sim \mathrm{N}\left[\left(\frac{1}{\sigma_i^2}\sum_{j=1}^{p}H_j^2+\frac{1}{\tau_\mu^2}\right)^{-1}\left(\frac{1}{\sigma_i^2}\sum_{j=1}^{p}H_j y_{ij}+\frac{1}{\tau_\mu^2}\sum_{k=1}^{m}S_{ik}\eta_k\right), \left(\frac{1}{\sigma_i^2}\sum_{j=1}^{p}H_j^2+\frac{1}{\tau_\mu^2}\right)^{-1}\right]$$

The full conditional of $\boldsymbol{\sigma}^2$ is given by

$$\begin{aligned}
\pi(\boldsymbol{\sigma}^2 \mid \bullet) &\propto \pi(\mathbf{Y}|\boldsymbol{\beta}, \boldsymbol{\mu}, \boldsymbol{\sigma}^2)\pi(\boldsymbol{\sigma}^2) \\
&\propto \prod_{i=1}^{q} \frac{1}{\sigma_i^p} \exp\left\{-\frac{1}{2\sigma_i^2} (\mathbf{y}_i - \mu_i \mathbf{H})^T (\mathbf{y}_i - \mu_i \mathbf{H})\right\} \times \frac{1}{\sigma_i^{2a_1+2}} \exp\left\{-\frac{a_2}{\sigma_i^2}\right\} \\
&\propto \prod_{i=1}^{q} \frac{1}{\sigma_i^{2a_1+p+2}} \exp\left\{-\frac{a_2 + \sum_{j=1}^{p}(y_{ij} - \mu_i H_j)^2/2}{\sigma_i^2}\right\}
\end{aligned}$$

This implies that we can update each σ_i^2 by sampling from

$$[\sigma_i^2 \mid \bullet] \sim \text{IG}\left[a_1 + \frac{p}{2}, a_2 + \frac{1}{2}\sum_{j=1}^{p}(y_{ij} - \mu_i H_j)^2\right]$$

The full conditional distribution of $\boldsymbol{\eta}$ is given by

$$\begin{aligned}
\pi(\boldsymbol{\eta} \mid \bullet) &\propto \pi(\boldsymbol{\mu}|\boldsymbol{\eta}, \tau_\mu^2)\pi(\boldsymbol{\eta}|\boldsymbol{\gamma}, \boldsymbol{\tau}^2) \\
&\propto \exp\left\{-\frac{1}{2\tau_\mu^2}(\boldsymbol{\mu} - \mathbf{S}\boldsymbol{\eta})^T(\boldsymbol{\mu} - \mathbf{S}\boldsymbol{\eta}) - \frac{1}{2}\boldsymbol{\eta}^T \boldsymbol{\Gamma}^{-1}\boldsymbol{\eta}\right\} \\
&\propto \exp\left\{-\frac{1}{2}\left[\boldsymbol{\eta}^T\left(\frac{1}{\tau_\mu^2}\mathbf{S}^T\mathbf{S} + \boldsymbol{\Gamma}^{-1}\right)\boldsymbol{\eta} - 2\left(\frac{\boldsymbol{\mu}^T\mathbf{S}}{\tau_\mu^2}\right)\boldsymbol{\eta}\right]\right\}
\end{aligned}$$

This implies that we can update $\boldsymbol{\eta}$ by sampling

$$[\boldsymbol{\eta} \mid \bullet] \sim \text{N}\left[\left(\frac{1}{\tau_\mu^2}\mathbf{S}^T\mathbf{S} + \boldsymbol{\Gamma}^{-1}\right)^{-1}\frac{\boldsymbol{\mu}^T\mathbf{S}}{\tau_\mu^2}, \left(\frac{1}{\tau_\mu^2}\mathbf{S}^T\mathbf{S} + \boldsymbol{\Gamma}^{-1}\right)^{-1}\right]$$

The full conditional distribution of $\boldsymbol{\gamma}$ is given by

$$\begin{aligned}
\pi(\boldsymbol{\gamma} \mid \bullet) &\propto \pi(\boldsymbol{\eta}|\boldsymbol{\gamma}, \boldsymbol{\tau}^2)\pi(\boldsymbol{\gamma}|w) \\
&\propto \prod_{k=1}^{m}\left[\frac{\omega_{0,k}}{\omega_{0,k} + \omega_{1,k}}\delta_{\nu_0} + \frac{\omega_{1,k}}{\omega_{0,k} + \omega_{1,k}}\delta_1\right],
\end{aligned}$$

where $\omega_{0,k} = (1-w)\nu_0^{-1/2}\exp\left(-\frac{\eta_k^2}{2\nu_0\tau_k^2}\right)$ and $\omega_{1,k} = w\exp\left(-\frac{\eta_k^2}{2\tau_k^2}\right)$.

We can update each γ_k by sampling from

$$[\gamma_k \mid \bullet] \sim \frac{\omega_{0,k}}{\omega_{0,k} + \omega_{1,k}}\delta_{\nu_0} + \frac{\omega_{1,k}}{\omega_{0,k} + \omega_{1,k}}\delta_1.$$

The full conditional of $\boldsymbol{\tau}^2$ is given by

$$\begin{aligned}\pi(\boldsymbol{\tau}^2 \mid \bullet) &\propto \pi(\boldsymbol{\eta}|\boldsymbol{\gamma}, \boldsymbol{\tau}^2)\pi(\boldsymbol{\tau}^2) \\ &\propto \prod_{k=1}^{m} \frac{1}{\tau_k} \exp\left\{-\frac{\eta_k^2}{2\tau_k^2\gamma_k}\right\} \times \frac{1}{\tau_k^{2c_1+2}} \exp\left\{-\frac{c_2}{\tau_k^2}\right\} \\ &\propto \prod_{k=1}^{m} \frac{1}{\tau_k^{2c_1+3}} \exp\left\{-\frac{c_2 + \eta_k^2/2\gamma_k}{\tau_k^2}\right\}.\end{aligned}$$

This implies that we can update each τ_k^2 by sampling

$$[\tau_k^2 \mid \bullet] \sim \text{IG}\left[c_1 + \frac{1}{2}, c_2 + \frac{\eta_k^2}{2\gamma_k}\right].$$

The full conditional of w is given by

$$\begin{aligned}\pi(w \mid \bullet) &\propto \pi(\boldsymbol{\gamma}|w)\pi(w) \\ &\propto \prod_{k=1}^{m} [(1-w)I[\gamma_k = \nu_0] + wI[\gamma_k = 1]] \\ &\propto w^{\sum_{k=1}^{m} I[\gamma_k=1]}(1-w)^{\sum_{k=1}^{m} I[\gamma_k=\nu_0]}.\end{aligned}$$

We update from

$$[w \mid \bullet] \sim \text{Beta}\left[1 + \sum_{k=1}^{m} I[\gamma_k = 1], 1 + \sum_{k=1}^{m} I[\gamma_k = \nu_0]\right].$$

The full conditional of τ_μ^2 is given by

$$\begin{aligned}\pi(\tau_\mu^2 \mid \bullet) &\propto \pi(\boldsymbol{\mu}|\boldsymbol{\eta}, \tau_\mu^2)\pi(\tau_\mu) \\ &\propto \frac{1}{\tau_\mu^q} \exp\left\{-\frac{1}{2\tau_\mu^2}(\boldsymbol{\mu} - \mathbf{S}\boldsymbol{\eta})^T(\boldsymbol{\mu} - \mathbf{S}\boldsymbol{\eta})\right\} \times \frac{1}{\tau_\mu^{2b_1+2}} \exp\left\{-\frac{b_2}{\tau_\mu^2}\right\} \\ &\propto \frac{1}{\tau_\mu^{2b_1+q+2}} \exp\left\{-\frac{b_2 + (\boldsymbol{\mu} - \mathbf{S}\boldsymbol{\eta})^T(\boldsymbol{\mu} - \mathbf{S}\boldsymbol{\eta})/2}{\tau_\mu^2}\right\}.\end{aligned}$$

Thus we sample from

$$[\tau_\mu^2 \mid \bullet] \sim \text{IG}\left[b_1 + \frac{q}{2}, b_2 + \frac{\sum_{i=1}^{q}(\mu_i - \mathbf{S}_i\boldsymbol{\eta})^T(\mu_i - \mathbf{S}_i\boldsymbol{\eta})}{2}\right].$$

1.2 Bayesian Level Set Methods with Normal Prior (Non Sparse Prior)

We also consider a conjugate normal prior on $\boldsymbol{\eta}$ without imposing sparsity which leads to more efficient posterior computation. The model is represented as

$$\begin{aligned}
&\mathbf{y}_i \mid \boldsymbol{\beta}, \mu_i, \sigma_i^2 \sim \mathrm{N}_p\left[\mu_i \mathbf{H}_\epsilon(\boldsymbol{\beta}), \sigma_i^2 \mathbf{I}_p\right],\ \boldsymbol{\beta} \sim \mathrm{N}_L\left[\mathbf{0}, \boldsymbol{\Lambda}_L\right], \\
&\boldsymbol{\mu} \sim \mathrm{N}_q[\mathbf{S}^T\boldsymbol{\eta}, \tau_\mu^2 \mathbf{I}_q],\ \boldsymbol{\eta} \sim \mathrm{N}_m[\mathbf{0}, \tau_\eta^2 \mathbf{I}_m], \\
&\sigma_i^2 \sim \mathrm{IG}[a_1, a_2],\ \tau_\mu^2 \sim \mathrm{IG}[b_1, b_2],\ \tau_\eta^2 \sim \mathrm{IG}[d_1, d_2].
\end{aligned}$$

The posterior computation algorithm for updating all other parameters remain the same except for $\boldsymbol{\eta}$ and τ_η^2, although we still use the Gibbs sampler to update these two parameters. We derive their full conditional distributions as follows.

The full conditional distribution of $\boldsymbol{\eta}$ is given by

$$\begin{aligned}
\pi(\boldsymbol{\eta} \mid \bullet) &\propto \pi(\boldsymbol{\mu}|\boldsymbol{\eta}, \tau_\mu^2)\pi(\boldsymbol{\eta}|\tau_\eta^2) \\
&\propto \exp\left\{-\frac{1}{2\tau_\mu^2}(\boldsymbol{\mu} - \mathbf{S}\boldsymbol{\eta})^T(\boldsymbol{\mu} - \mathbf{S}\boldsymbol{\eta}) - \frac{1}{2\tau_\eta^2}\boldsymbol{\eta}^T\boldsymbol{\eta}\right\} \\
&\propto \exp\left\{-\frac{1}{2}\left[\boldsymbol{\eta}^T\left(\frac{1}{\tau_\mu^2}\mathbf{S}^T\mathbf{S} + \frac{1}{\tau_\eta^2}\mathbf{I}_m\right)\boldsymbol{\eta} - 2\left(\frac{\boldsymbol{\mu}^T\mathbf{S}}{\tau_\mu^2}\right)\boldsymbol{\eta}\right]\right\}.
\end{aligned}$$

This implies that we can update $\boldsymbol{\eta}$ by sampling

$$[\boldsymbol{\eta} \mid \bullet] \sim \mathrm{N}\left[\left(\frac{1}{\tau_\mu^2}\mathbf{S}^T\mathbf{S} + \frac{1}{\tau_\eta^2}\mathbf{I}_m\right)^{-1}\frac{\boldsymbol{\mu}^T\mathbf{S}}{\tau_\mu^2}, \left(\frac{1}{\tau_\mu^2}\mathbf{S}^T\mathbf{S} + \frac{1}{\tau_\eta^2}\mathbf{I}_m\right)^{-1}\right].$$

The full conditional of τ_η^2 is given by

$$\begin{aligned}
\pi(\tau_\eta^2 \mid \bullet) &\propto \pi(\boldsymbol{\eta}|\tau_\eta^2)\pi(\tau_\eta^2) \\
&\propto \frac{1}{\tau_\eta^m}\exp\left\{-\frac{1}{2\tau_\eta^2}\boldsymbol{\eta}^T\boldsymbol{\eta}\right\} \times \frac{1}{\tau_\eta^{2d_1+2}}\exp\left\{-\frac{d_2}{\tau_\eta^2}\right\} \\
&\propto \frac{1}{\tau_\eta^{2d_1+m+2}}\exp\left\{-\frac{d_2 + \boldsymbol{\eta}^T\boldsymbol{\eta}/2}{\tau_\eta^2}\right\}.
\end{aligned}$$

Thus we sample from

$$[\tau_\eta^2 \mid \bullet] \sim \mathrm{IG}\left[d_1 + \frac{m}{2}, d_2 + \frac{\sum_{k=1}^m \eta_k^2}{2}\right].$$
